# Strong Coupling of Shoot Assimilation and Soil Respiration during Drought and Recovery Periods in Beech As Indicated by Natural Abundance δ^13^C Measurements

**DOI:** 10.3389/fpls.2016.01710

**Published:** 2016-11-17

**Authors:** Carola H. Blessing, Matti Barthel, Lydia Gentsch, Nina Buchmann

**Affiliations:** ^1^Centre for Carbon Water and Food, University of Sydney, Brownlow HillNSW, Australia; ^2^Institute of Agricultural Sciences, ETH ZürichZürich, Switzerland; ^3^Chair of Bioclimatology, Georg-August University of GöttingenGöttingen, Germany

**Keywords:** carbon allocation, soil CO_2_ eﬄux, drought, beech (*Fagus sylvatica*), laser spectroscopy, resilience

## Abstract

Drought down-regulates above- and belowground carbon fluxes, however, the resilience of trees to drought will also depend on the speed and magnitude of recovery of these above- and belowground fluxes after re-wetting. Carbon isotope composition of above- and belowground carbon fluxes at natural abundance provides a methodological approach to study the coupling between photosynthesis and soil respiration (SR) under conditions (such as drought) that influence photosynthetic carbon isotope discrimination. In turn, the direct supply of root respiration with recent photoassimilates will impact on the carbon isotope composition of soil-respired CO_2_. We independently measured shoot and soil CO_2_ fluxes of beech saplings (*Fagus sylvatica* L.) and their respective δ^13^C continuously with laser spectroscopy at natural abundance. We quantified the speed of recovery of drought stressed trees after re-watering and traced photosynthetic carbon isotope signal in the carbon isotope composition of soil-respired CO_2_. Stomatal conductance responded strongly to the moderate drought (-65%), induced by reduced soil moisture content as well as increased vapor pressure deficit. Simultaneously, carbon isotope discrimination decreased by 8‰, which in turn caused a significant increase in δ^13^C of recent metabolites (1.5–2.5‰) and in δ^13^C of SR (1–1.5‰). Generally, shoot and soil CO_2_ fluxes and their δ^13^C were in alignment during drought and subsequent stress release, clearly demonstrating a permanent dependence of root respiration on recently fixed photoassimilates, rather than on older reserves. After re-watering, the drought signal persisted longer in δ^13^C of the water soluble fraction that integrates multiple metabolites (soluble sugars, amino acids, organic acids) than in the neutral fraction which represents most recently assimilated sugars or in the δ^13^C of SR. Nevertheless, full recovery of all aboveground physiological variables was reached within 4 days – and within 7 days for SR – indicating high resilience of (young) beech against moderate drought.

## Introduction

In trees, root growth and respiration is fuelled by a rapid transport of recent photoassimilates from leaves to roots (Brüggemann et al., 2011; Epron et al., 2012). This causal relationship between above- and belowground physiological processes – often termed aboveground to belowground coupling – has been shown by girdling and stable isotope tracer studies (Högberg et al., 2001; Kuzyakov and Gavrichkova, 2010). For beech trees, transport velocities of up to 1 m h^-1^ have been reported (Plain et al., 2009; Gavrichkova et al., 2011; Kuptz et al., 2011), depending on seasonality (Kuptz et al., 2011), tree height (Kuzyakov and Gavrichkova, 2010), and environmental conditions like drought (Barthel et al., 2011). In a forest ecosystem, complexity is added to the straightforward link between assimilatory and respiratory CO_2_ fluxes from the soil, e.g., due to different sources contributing to soil CO_2_ fluxes (microbial vs. plant-related), different plant species and plant ages as well as changing environmental conditions on diurnal and seasonal scales. However, as forests are an important terrestrial carbon sink (Pan et al., 2011), it is important to understand the dynamics of these opposing CO_2_ fluxes, especially because ecosystem perturbations are predicted to occur more frequently.

Drought is a major climatic factor determining carbon and water fluxes (Granier et al., 2007), thus productivity in forest ecosystems (Breda et al., 2006), and gains increased importance under future climatic conditions (Schär et al., 2004; Reichstein et al., 2013). Beech, an important forest tree species in Central Europe, is characterized as drought sensitive, and is predicted to suffer during extreme drought events (Granier et al., 2007), both in terms of growth and competitive ability (Gessler et al., 2007b). However, recent studies showed that beech tolerates moderate drought events, as it maintains allocation of recent photoassimilates into the roots (Blessing et al., 2015; Scartazza et al., 2015; Hommel et al., 2016). Thus, studying the effect of drought on carbon and water fluxes will provide crucial insights into the capability of European beech forests to cope with more frequent drought spells in the future.

Moreover, the drought sensitivity of beech trees will not only depend on how strongly they are affected by drought itself, but also on their ability to recover after the drought stress has been released (Zang et al., 2014), i.e., on their resilience (Grimm and Wissel, 1997). Watering beech after a drought spell showed that trees are capable to immediately utilize the supplied water (Barthel et al., 2014; Volkmann et al., 2016). A fast recovery of a plant’s water status will help to retrieve full physiological activity in form of carbon and water fluxes. However, above- and belowground carbon and water fluxes may differ in their speed and magnitude of recovery after rewetting, therefore a separate analysis of both helps to understand their impact on the ecosystem level.

Studies on the coupling of above- to belowground carbon fluxes at natural carbon isotope abundance use variations in carbon discrimination Δ^13^C, which is dependent on the ratio of the CO_2_ concentration in the intercellular air space to the atmospheric CO_2_ concentration (C_i_/C_a_) (Farquhar et al., 1982). C_i_/C_a_ in turn is modified by environmental conditions (Cernusak et al., 2013). Multiple studies could track the imprint of Δ^13^C in a time lagged carbon isotope signature in stem phloem (e.g., Keitel et al., 2003; Merchant et al., 2010; Rascher et al., 2010), root phloem (Scartazza et al., 2015), soil respiration (SR; Ekblad and Högberg, 2001; McDowell et al., 2004), and ecosystem respiration (McDowell et al., 2004; Knohl et al., 2005; Werner et al., 2006; Alstad et al., 2007). These measurable changes in the carbon isotope composition of soil and ecosystem respired CO_2_ were detected using discrete instead of continuous isotope measurements. However, first *in situ* real-time measurements revealed that such conclusions are not that straightforward as Δ^13^C displays diurnals changes (Gentsch et al., 2014) which are not always reflected in the carbon isotope signature of SR, especially under wet conditions (Wingate et al., 2010).

A process-based understanding, both of the dynamics in the opposing ecosystem CO_2_ fluxes and their respective carbon isotopic signatures, cannot be attained solely by ecosystem-scale studies or by studying single ecosystem components. Therefore, simple small-scale model ecosystems may help to arrive at a better understanding of how a change in canopy assimilation confers to changes in SR, and respective carbon isotope signatures. At the same time, such systems have the advantage of providing controlled and stable conditions, helping to infer underlying processes. Here we present a drought experiment with beech saplings. We followed the coupling between above- and belowground fluxes and their carbon isotope composition at natural abundance levels also into the – often neglected – recovery period, using *in situ* real-time laser spectroscopy measurements in shoot and soil CO_2_ fluxes over about 1 month.

Using a simple controlled system, we asked the following questions:

(a) How quickly do plant physiological variables (i.e., stomatal conductance, photosynthesis, transpiration), assimilatory and respiratory CO_2_ fluxes recover from the induced drought spell after re-watering in our beech model ecosystem?
(b) Is the expected large change in photosynthetic carbon isotope discrimination under drought and during recovery traceable to a prominent change in belowground carbon isotope composition of soil-respired CO_2_? And vice versa, is it feasible to draw conclusions from the carbon isotope composition of soil-respired CO_2_ also at natural abundance on the influence of plant physiological variables?

We focus on the short term response of our drought stressed beech model ecosystem to re-watering. Since recently fixed assimilates fuel root respiration to a high extent, we expect a prominent change in the observed carbon isotope discrimination during drought, with more ^13^C enriched photoassimilates imprinting on the δ^13^C of root and thus SR. We further expect that SR increases after re-watering and its carbon isotope signal becomes more depleted in ^13^C due to increased Δ^13^C after stress release with a delay to aboveground variables due to the transport time of photoassimilates from the leaves to the roots and back into the atmosphere.

## Materials and Methods

### Plant Material

Six 0.8 m tall beech saplings were enclosed individually in custom-made soil-shoot chambers that separately enclosed shoot and soil compartments of one beech sapling as described in Barthel et al. (2011). Each soil-shoot chamber was equipped with sensors for relative humidity (Hygroclip^®^ S3C03, rotronic AG, Bassersdorf, Switzerland), soil moisture (ECH_2_O EC-5, Decagon Devices, Inc., Pullman, WA, USA) as well as air (Hygroclip^®^ S3C03, rotronic AG, Bassersdorf, Switzerland), leaf (Thermocouple Type K, Omega Enginerring Inc., Stamford, CT, USA), and soil (AD 592, Analog Devices Inc., Norwood, MA, USA) temperatures. In addition, each shoot chamber was equipped with a small fan to ensure homogeneous air mixing within the shoot compartment. All six soil-shoot chambers were placed inside a climate chamber controlling the day-night cycle of 16/8 h for temperature and light intensity inside the soil-shoot chambers. Light intensity steadily increased or decreased during the first 3 and last 5 hours of the daytime period; so plants received maximal photosynthetic active radiation of about 600 μmol m^-2^ s^-1^ between 10 am and 6 pm.

Beech saplings grown in 7.9 l pots with potting soil (Containererde, Ökohum, Herrenhof, Switzerland) had fully developed leaves in April, when the experiment started (bud burst in February). They were irrigated daily via tubing connected to the soil chambers, replacing the amount of water lost via transpiration and via evaporation calculated from the previous day gas exchange measurements. For the experiment, the saplings were separated into two groups of three beech saplings each which experienced a different water treatment. One group served as control, for which daily irrigation was continued during the whole experiment (04 April 2009 – 05 May 2009, DOY 94–125). The other group, however, was subject to, first, reduced daily irrigation (9 April 2009, DOY 99; drought period) and, then, re-watering (26 April 2009 2:30 am, DOY 116) during the night in order to allow saturation of soil water and plant tissues (recovery period). In the following, this group is referred to as “drought-treated” saplings. The drought treatment affected both soil moisture content (SMC) and vapor pressure deficit (VPD), being dependent of the transpiration rate. We refer to DOY 116 as the first day after re-watering since re-watering took place at pre-dawn of this day. Drought-treated saplings showed significantly lower soil moisture than control saplings from 14 April 2009 (DOY 104) onward, therefore we focus on data after this day. The main drought period, during which assimilation and stomatal conductance showed a significant effect to the drought treatment, lasted from 17 April 2009 (DOY 107) until re-watering.

### Continuous Gas-Exchange and Carbon Isotope Measurements with Laser Spectroscopy

The gas-tight separation between shoot and soil, realized by the combined soil-shoot chamber system, enabled independent measurements of above- and belowground gas exchange and the carbon isotopic composition of CO_2_. By continuously flushing the chambers with a vacuum pump (VTE 6 for soil chambers/VTL 15 for shoot chambers, Gardner Denver Inc., Quincy, IL, USA), steady-state conditions were achieved. The flow rate was adjusted according to the gas exchange of the shoot or soil during the day, to avoid condensation and to obtain sufficient differences in CO_2_ concentrations between inlet and outlet (shoot chamber 12–16 l min^-1^, soil chamber 2.1–2.3 l min^-1^), but then kept constant during the whole experiment. A valve system alternately directed a subsample of 0.5 l min^-1^ of the inlet air (surrounding air inside the climate chamber) and of the outlet air of one soil-shoot chamber for 134 and 136 s, respectively, to a pulsed quantum cascade absorption laser spectrometer (QCLAS-ISO, Aerodyne Research, Inc., Billerica, MA, USA). The laser spectrometer, located outside of the climate chamber, was used to simultaneously measure the CO_2_ isotopologues ^12^C^16^O_2_, ^13^C^16^O_2_, and ^12^C^16^O^18^O at a rate of 0.5 Hz by scanning across three spectral lines near 4.3 μm (2310 cm^-1^). The measurement principle is based upon two optical multipass absorption cells with stabilized pressure and temperature using a spectral ratio method (Nelson et al., 2008). Throughout the whole experiment, calibration was done every hour for 6 min in three consecutive steps, including dilution calibration, span calibration, and check for long-term calibration stability (Sturm and Knohl, 2010). The performed calibration resulted in an Allan deviation of 0.25‰ at 1 s averaging time for δ^13^C. Further, water vapor concentrations were measured with an infrared gas analyzer system (IRGA; Li6262, Li-Cor Biosciences Inc., Lincoln, NE, USA). Real-time data acquisition/processing and controlling of instruments, calibration units, chambers, valves, and sensors were realized by a custom-written LabVIEW program (LabVIEW, National Instruments Corp., Austin, TX, USA). The first 20 s of each sampling interval were excluded for average calculation due to the time lag after valve switching. The outlets of the shoot and the soil chambers of one sapling subjected to drought and of one control sapling were measured in alternating mode.

### Leaf Sampling and δ^13^C Analysis

Three to five leaves per replicated beech sapling were sampled in order to determine the δ^13^C values of leaf material during the period of drought stress (23 April 2009, DOY 113) and at the end of the experiment during the recovery period (05 May 2009, DOY 125). Leaf samples were immediately put in liquid nitrogen after sampling and then kept frozen at -20°C until leaf material was homogenized under addition of liquid nitrogen with mortar and pestle. For δ^13^C analysis of bulk leaf material, a subsample was dried at 60°C for 3 days and the milled powder was weighed into tin capsules for isotope ratio mass spectrometry (IRMS) analysis (MAT Delta^plus^XP, Finnigan MAT, Bremen, Germany).

For the extraction of water soluble and the neutral organic carbon fractions, 100 mg of the remaining homogenized leaf material was then suspended in 1 ml de-mineralized water and put on ice for 60 min. Afterward, samples were kept at 100°C for 3 min in order to precipitate proteins (Gessler et al., 2007a). The solution was then centrifuged at 12000 × *g*. A subsample was transferred into tin capsules and dried in order to analyze δ^13^C of the water soluble organic carbon fraction (δ^13^C_ws_). The remaining supernatant was mixed with anion and cation exchange resins (Dowex 1 and Dowex 50, VWR International AG, West Chester, PA, USA) in order to remove amino acids and organic acids (Göttlicher et al., 2006). The final solution was then pipetted into tin capsules, dried and analyzed for δ^13^C of the neutral organic carbon fraction (δ^13^C_nf_). This neutral fraction represents mainly recently assimilated soluble sugars (Göttlicher et al., 2006), whereas the water soluble fraction includes also other compounds such as soluble amino acids and organic acids (Gessler et al., 2007a). IRMS analysis followed the identical treatment principle described in Werner and Brand (2001). The standard deviation of the long-term quality control standard (tyrosine) was 0.09‰ (over 9 years). In addition, on 05 May 2009 (DOY 125), all remaining leaves were collected to determine overall leaf area per plant (Li3000, Li-Cor Biosciences Inc., Lincoln, NE, USA).

### Calculation of Carbon Fluxes and the Corresponding Isotope Ratios

Stomatal conductance (g_s_), transpiration (E), and net shoot assimilation (A_N_) were calculated according to von Caemmerer and Farquhar (1981), while the calculation of SR followed the calculations presented in Burri et al. (2014).

All carbon isotope values are reported on the Vienna-Pee Dee Belemnite (V-PDB) scale using the δ-notation:

(1) $$\begin{array}{c} \;\delta^{13}C=\frac{R_{\mathrm{sample}}}{R_{V-PDB}}-1\;\;\;\;[\%], \end{array}$$

where *R*_sample_ and *R*_V -PDB_ denote the ^13^C/^12^C ratios of the sample and the standard, respectively. The method after Evans et al. (1986) was used to calculate on-line photosynthetic carbon isotope discrimination (Δ^13^C_obs_) as follows:

(2) $$\begin{array}{c} \Delta^{13}C_{\mathrm{obs}}=\frac{\xi \cdot (\delta^{13}C_{\mathrm{out}}-\delta^{13}C_{\mathrm{in}})}{1+\;\delta^{13}C_{\mathrm{out}}-\xi \cdot (\delta^{13}C_{\mathrm{out}}-\delta^{13}C_{\mathrm{in}})}, \end{array}$$

where ξ denotes the ratio of CO_2_ entering the chamber in relation to the photosynthetic flux [c_in_/(c_in_-c_out_)]. Further, δ^13^C_in_ and δ^13^C_out_ denote the respective carbon isotopic compositions of CO_2_ entering and leaving the chamber, respectively.

The δ^13^C signal of SR (δ^13^C_SR_) was calculated using an isotopic mass balance equation:

(3) $$\begin{array}{c} \;\delta^{13}C_{\mathrm{SR}}=\frac{\;\delta^{13}C_{\mathrm{out}}\cdot c_{\mathrm{out}}-\delta^{13}C_{\mathrm{in}}\cdot c_{\mathrm{in}}}{c_{\mathrm{out}}-c_{\mathrm{in}}}, \end{array}$$

where c_in_ and c_out_ refer to CO_2_ mole fractions at chamber inlet and outlet, respectively.

### Statistics

The relative effect of drought was expressed as drought effect, i.e., (100*treatment/control)-100. Full recovery from drought stress was achieved when the mean drought effect plus standard deviation exceeded 100%, and was represented in days after re-watering, with day one referring to DOY 116. Standard deviations of differences between control and drought-treated saplings are error propagated as follows:

(4) $$\begin{array}{c} \;SD_{\mathrm{diff}}=\sqrt{SD_{\mathrm{wet}}^{2}+SD_{\mathrm{dry}}^{2}} \end{array}$$

with SD_wet_ and SD_dry_ representing hourly standard deviations of control and drought-treated samplings (*n* = 3).

The drought effect on measured plant physiological variables and isotopic signatures was tested with a linear mixed effect model using the nlme-package (Pinheiro et al., 2016) and the statistical software R [Version 3.0.3; R Development Core Team (2008–2010)], including the beech sapling as random effect in the model. If not noted otherwise, day refers to a light intensity above 500 μmol m^-2^ s^-1^, and night to a light intensity below 20 μmol m^-2^ s^-1^. As normal distribution cannot be tested based on three samples, a Wilcoxon–Mann–Whitney test (Wilcox test in R) was used for δ^13^C values of samples measured with the IRMS. The relationship between diurnal mean values of A_N_, E, and gs to SR (PAR > 500 μmol m^-2^ s^-1^), as well as the relationship between Δ^13^C_obs_ and δ^13^C_SR_ were tested using repeated measure analysis (nlme-package).

## Results

### Environmental Conditions

**FIGURE 1:**
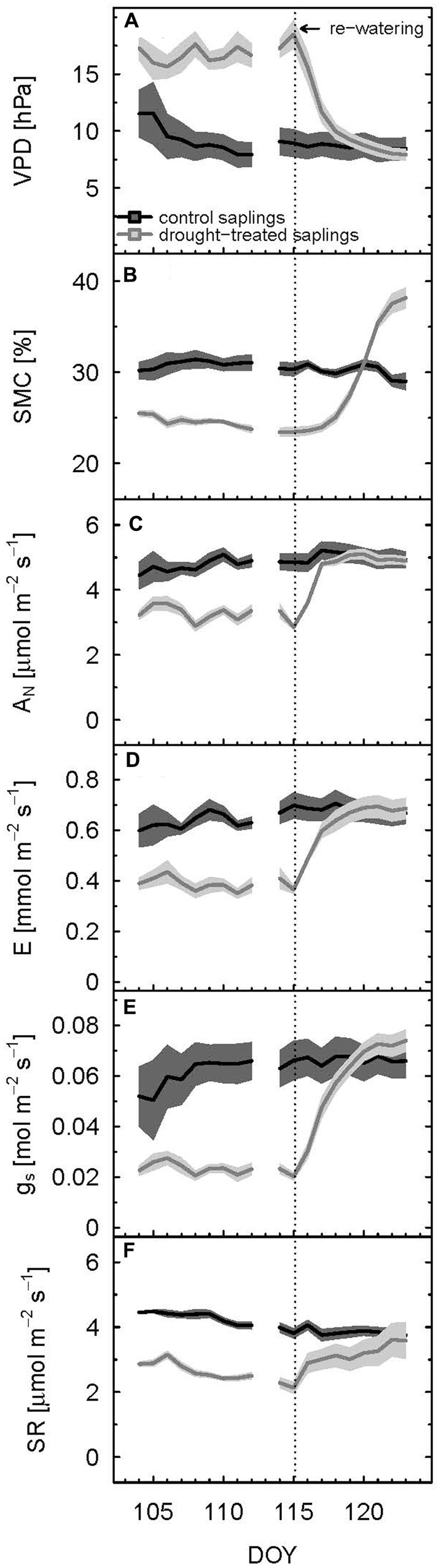
**Diurnal means of vapor pressure deficit (VPD) (A)**, soil moisture content **(B)**, net assimilation **(C)**, transpiration **(D)**, stomatal conductance to H_2_O **(E)**, and soil respiration **(F)** of control and drought-treated beech saplings. Mean values were calculated for PAR > 500 μmol m^-2^ s^-1^. The shaded areas show propagated standard deviations of three replicates. Re-watering is indicated by the dashed vertical line.

**FIGURE 2:**
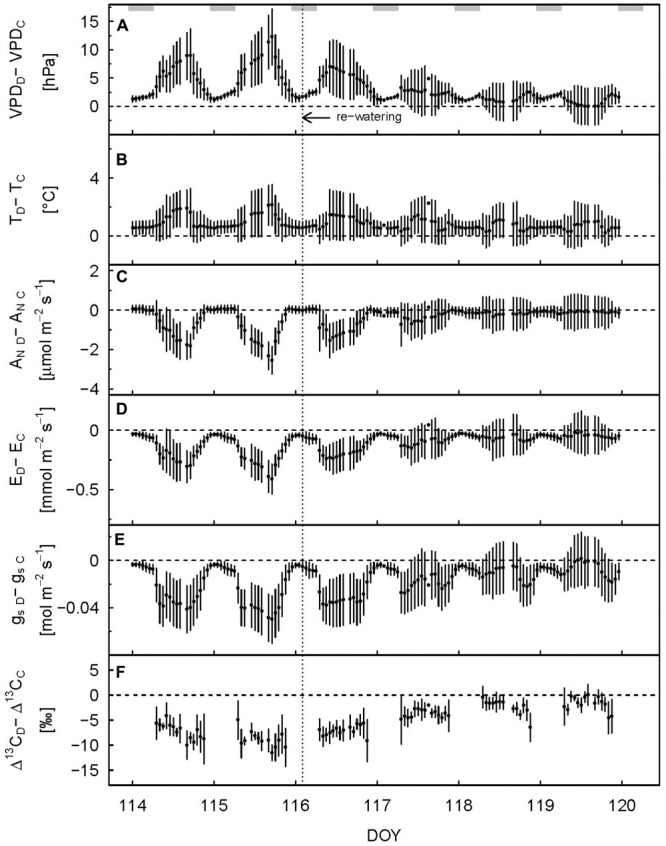
**Hourly difference in vapor pressure deficit (A)**, air temperature **(B)**, net assimilation **(C)**, transpiration **(D)**, stomatal conductance to H_2_O **(E)**, and observed photosynthetic carbon isotope discrimination **(F)** between drought-treated (subscript D) and control (subscript C) saplings 2 days before and 4 days after re-watering. Shaded rectangles at the top indicate nighttime. Re-watering is represented by the dashed vertical line. Error bars show error propagated standard deviation, data are only shown when at least two replicates were available.

During the main drought period (DOY 107–115), VPD approximately doubled during the daytime for drought-treated compared to control plants, reaching an average of 17.1 hPa for the entire drought period (**Table 1**; **Figure 1A**). In contrast, air, leaf, and soil temperatures were not significantly affected by the drought treatment. Air temperature during the drought period was slightly lower for control saplings compared to droughttreated saplings (**Figure 2B**) with mean values of 25.4 and 26.7°C, respectively, and night air temperatures were 17.2 and 17.8°C, respectively. Average leaf temperatures were 1–2°C below air temperature. SMC decreased significantly from 31 to 24% for control and drought-treated saplings, respectively (**Table 1**).

**Table 1:** Mean values ± propagated standard deviations for environmental variables (vapor pressure deficit, air temperature, leaf temperature, soil temperature, and soil moisture), gas exchange variables (net assimilation, stomatal conductance to H_2_O, transpiration, and soil respiration), observed photosynthetic discrimination and the δ^13^C of soil-respired CO_2_ for day time (PAR > 500 μmol m^-2^ s^-1^) of the entire drought period (17–25 April, DOY 107–115) of the control and the drought treatment.

	Control	Drought-treated	Drought effect	*p*-value
VPD [hPa]	8.7 ± 0.43	17.1 ± 0.38	96.9%	**0.023**
Air temperature [°C]	25.4 ± 0.03	26.7 ± 0.18	5.0%	0.147
Leaf temperature [°C]	24.6 ± 0.18	24.7 ± 0.24	0.5%	0.876
Soil temperature [°C]	16.7 ± 0.08	17.1 ± 0.11	2.3%	0.747
Soil moisture [%]	30.9 ± 0.28	24.1 ± 0.14	-21.9%	**0.008**
A_N_ [μmol m^-2^ s^-1^]	4.84 ± 0.08	3.19 ± 0.06	-34.1%	**0.017**
g_s_ H_2_O [mol m^-2^ s^-1^]	0.06 ± 0.00	0.02 ± 0.00	-64.9%	**0.017**
E [mmol m^-2^ s^-1^]	0.65 ± 0.01	0.38 ± 0.01	-42.0%	**0.020**
Δ^13^C_obs_ [‰]	24.90 ± 0.16	17.05 ± 0.17	-31.5%	**<0.001**
SR day [μmol m^-2^ s^-1^]	4.16 ± 0.04	2.46 ± 0.04	-40.8%	**0.003**
δ^13^C_SR_ day [‰]	-25.52 ± 0.09	-24.37 ± 0.10	-4.5%	**0.008**
SR night [μmol m^-2^ s^-1^]	4.25 ± 0.05	2.16 ± 0.06	-49.3%	**0.005**
δ^13^C_SR_ night [‰]	-25.97 ± 0.07	-24.57 ± 0.17	-5.4%	**0.013**

*The drought effect was calculated from mean values of control and drought-treated saplings for the entire drought period for each environmental variable. *P*-values (significant *p* < 0.05 formatted in bold) arise from a linear mixed model including the treatment as fixed and each sapling as random effect.*

After re-watering the drought-treated saplings, VPD and SMC approximated the respective values of the control saplings, and even exceeded control values for SMC (**Figures 1A,B**). Mean VPD in the shoot chamber of drought-treated saplings declined slightly below those of the control 5 days after re-watering (DOY 120), but stayed in a range of about 10% deviation. In contrast, SMC of drought-treated saplings exceeded that of the control by about 30% toward the end of the experiment (DOY 123), which showed that the soil of control saplings was not fully saturated. Note, this was done on purpose to avoid anaerobic soil conditions during the experiment.

### Carbon Dioxide and Water Vapor Fluxes: Magnitude and Carbon Isotope Composition

#### Drought

We defined the main drought period from DOY 107–115, as the drought treatment showed a significant effect on diurnal assimilation and stomatal conductance during these days (*p* < 0.05, linear mixed model). Stomatal conductance (g_s_; during the day) was most sensitive to drought stress. On average, it decreased by 65% (**Table 1**; **Figure 1E**) in drought-treated saplings compared to control saplings, while transpiration (E; **Figure 1D**) and net assimilation (A_N_; **Figure 1C**) decreased by 42 and 34%, respectively. While control plants showed relatively stable A_N_, E, and g_s_ during full illumination (**Figures 1C–E**), these plant physiological variables decreased during the day in drought-treated plants, leading to an increasing difference between drought-treated and control saplings (**Figures 2C**–**E**). Simultaneously, VPD steadily increased during the day (**Figure 2A**).

**FIGURE 3:**
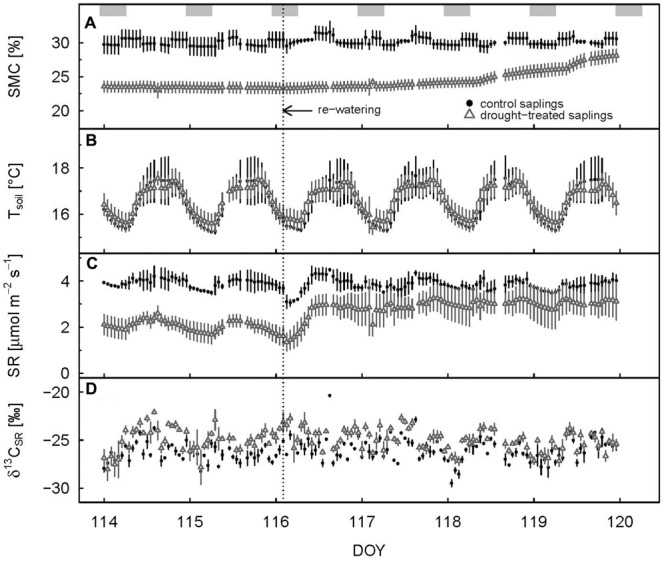
**Hourly mean values of soil moisture (A)**, soil temperature **(B)**, soil respiration **(C)**, and δ^13^C value of soil respiration **(D)** for control and drought-treated saplings 2 days before and 4 days after re-watering. Shaded rectangles at the top indicate nighttime. Re-watering is represented by the dashed vertical line. Error bars show the standard errors; data are only shown when at least two replicates were available.

Soil respiration of drought-treated saplings was reduced between 40 and 50% compared to the control, both during day (PAR > 500 μmol m^-2^ s^-1^) and night (PAR < 20 μmol m^-2^ s^-1^; **Table 1**; **Figure 1F**). Thus, drought reduced SR more than net assimilation. During the drought period, SR of control saplings remained constantly higher than that of drought-treated saplings, with little variation between day and night (**Figure 3C**). Mean daytime values of SR was 4.2 and 2.5 μmol m^-2^ s^-1^ for control and drought-treated saplings, respectively, while nighttime SR was 4.3 and 2.2 μmol m^-2^ s^-1^ for control and drought-treated saplings, respectively.

**FIGURE 4:**
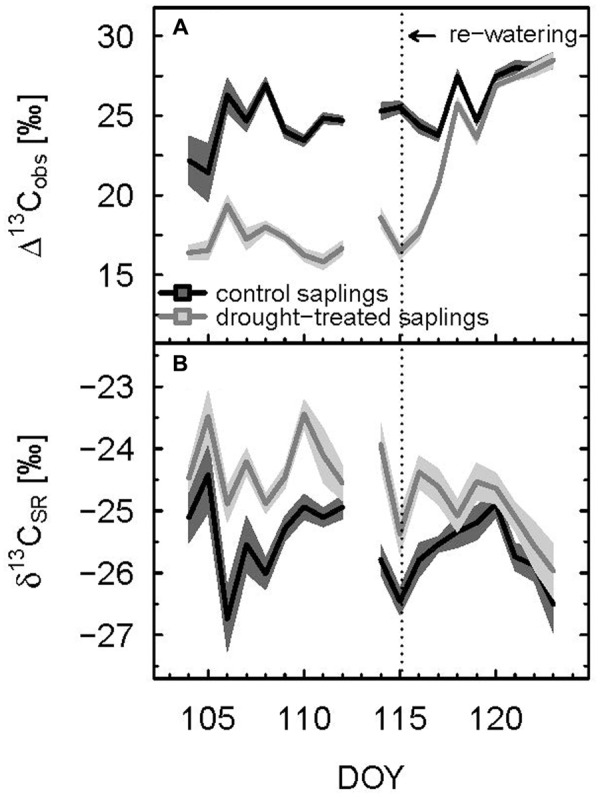
**Diurnal means of observed photosynthetic carbon isotope discrimination (A)** and δ^13^C value of soil respiration **(B)** of control and drought-treated beech saplings. Mean values were calculated for PAR > 500 μmol m^-2^ s^-1^. The shaded areas show propagated standard deviations of three replicates. Re-watering is indicated by the dashed vertical line.

Δ^13^C_obs_ showed a relatively large day-to-day variability, even for control plants (**Figure 4A**) Average shoot Δ^13^C_obs_ significantly decreased from 24.9 to 17.1‰ during the drought period (*p* < 0.001; **Table 1**; **Figure 4B**), which resulted in a corresponding ^13^C enrichment of recent assimilates in leaves, i.e., larger δ^13^C_ws_ and δ^13^C_nf_ during drought (*p*-values = 0.05; **Table 2**). The enrichment in ^13^C was larger in the neutral fraction (2.49‰) compared to the water soluble fraction (1.42‰). Bulk leaf δ^13^C_bulk_ of control and drought-treated saplings, however, did not differ (*p* = 0.5). The δ^13^C values of soil-respired CO_2_ (δ^13^C_SR_) of control plants were similar during day and night, with on average -25.5 and -26.0‰, respectively (**Table 1**). Drought caused a significant increase of δ^13^C_SR_ by about 1‰ during the day and about 1.5‰ during the night (*p* = 0.008 for day and 0.013 for night; **Table 1**; **Figures 3D** and **4B**).

**Table 2:** Mean values ± standard errors of δ^13^C values of leaf bulk organic material, water soluble (ws) and neutral (nf) fractions.

		Control	Drought-treated	*p*-value
Drought period	δ^13^C_bulk_	-29.00 ± 0.23	-28.98 ± 0.09	0.500
	δ^13^C_ws_	-28.63 ± 0.25	-27.21 ± 0.08	0.050
	δ^13^C_nf_	-28.04 ± 0.23	-25.55 ± 0.27	0.050
Recovery period	δ^13^C_bulk_	-29.17 ± 0.25	-29.12 ± 0.16	0.500
	δ^13^C_ws_	-29.55 ± 0.14	-28.81 ± 0.20	0.050
	δ^13^C_nf_	-29.12 ± 0.02	-28.58 ± 0.58	0.350

*Sampling was at the end of the drought period (23 April 2009, DOY 113) and 10 days after re-watering (05 May 2009, DOY 125). *P*-values arise from a Wilcoxon test testing control vs. drought-treated δ^13^C values.*

#### Recovery Period

Full recovery was achieved within a few days after re-watering for gas exchange, with A_N_ recovering first, then g_s_ and E, and finally SR (**Figure 1**). After 2 days (DOY 117), A_N_ reached the 90% recovery level, and after 3 days (DOY 118), it was fully recovered (mean plus standard deviation > 100%) (**Figure 2C**). Mean recovery level of transpiration was 88% 2 days after re-watering (DOY 117) and full recovery level was reached on DOY 119 (**Figure 2D**). Similarly, stomatal conductance fully recovered 4 days after re-watering (DOY 119), but only recovered to 74% of the control level 2 days after re-watering (DOY 117) (**Figure 2E**). SR showed a strong initial recovery rate at the day of re-watering (**Figures 1F** and **3C**), which later leveled off to a moderate recovery rate. Full recovery of SR took 7 days (DOY 122; **Figure 1**), during which also the amplitude of its diurnal cycle diminished (**Figure 3**).

Δ^13^C_obs_ reached the 90% level of recovery, which means a difference of less than 2.75‰, 3 days after re-watering (DOY 118; **Figure 2F**) and full recovery 7 days after re-watering (DOY 122; **Figure 4A**). δ^13^C_ws_ values of leaf material sampled 10 days after re-watering (DOY 125) still showed a treatment effect (*p*-value = 0.05), in contrast to δ^13^C of the neutral fraction, although the standard errors for mean δ^13^C_nf_ of drought-treated saplings were much larger compared to those of control saplings (**Table 2**). The δ^13^C signal of SR approached full recovery within 3 days (DOY 118) after re-watering (**Figures 3D and 4B**), but due to variability between days, mean diurnal values of δ^13^C_SR_ were still below control on some days afterward. Therefore, it took until DOY 122 until full recovery of δ^13^C_SR_ remained for two consecutive days, thus at the same time as Δ^13^C_obs_.

### Coupling of Above and Belowground Carbon Fluxes

**FIGURE 5:**
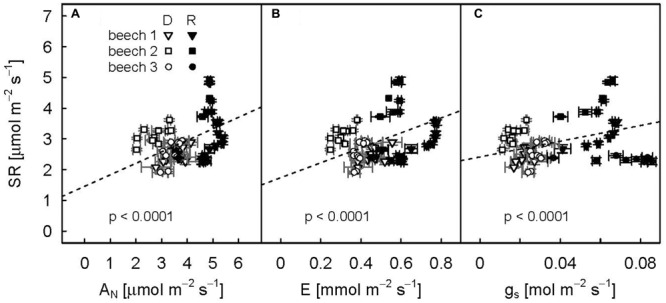
**Relationships between diurnal means of net assimilation (A)**, transpiration **(B)**, and stomatal conductance **(C)** and soil respiration for the three drought-treated beech saplings. The drought period (DOY 104–115) is represented by open symbols, the recovery period (DOY 116–124) by filled symbols. Diurnal means and standard deviations (error bars) are calculated for PAR > 500 μmol m^-2^ s^-1^. Repeated measures analysis for all relationships were significant (*p* values < 0.0001, dashed line).

Even though SR varied considerably between individual drought-treated plants especially during the recovery period, repeated measure analysis showed a strong positive relation (*p*-values < 0.0001) between diurnal mean SR and aboveground plant physiological variables (**Figure 5**; trend lines given).

**FIGURE 6:**
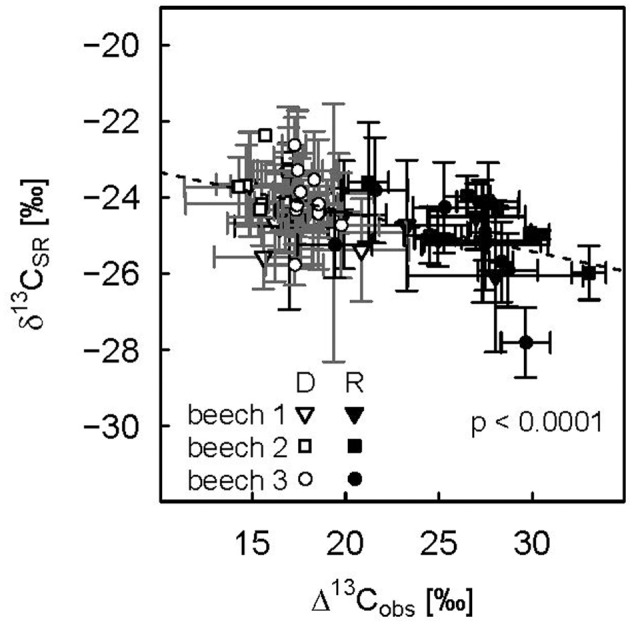
**Relationship between diurnal means of photosynthetic discrimination and the δ^13^C value of soil respiration for the three drought-treated beech saplings.** The drought period (DOY 104–115) is represented by open symbols, the recovery period (DOY 116–124) by filled symbols. Diurnal means and standard deviations (error bars) are calculated for PAR > 500 μmol m^-2^ s^-1^. Repeated measures analysis was significant (*p* values < 0.0001, dashed line).

Lower Δ^13^C_obs_ indicating more ^13^C enriched photoassimilates during the drought period was reflected in less negative δ^13^C_SR_, while increasing Δ^13^C_obs_ during the recovery period lead to a direct decrease in diurnal mean δ^13^C_SR_ (**Figure 6**). This dependency was reflected in a significant negative relationship between Δ^13^C_obs_ and δ^13^C_SR_ of drought-treated plants. Overall, Δ^13^C_obs_ differed about 15‰ between drought and recovery periods for drought-treated plants, while δ^13^C_SR_ only spanned about 2‰.

## Discussion

### Drought Effect on Soil Respiration and δ^13^C_SR_ at Natural Abundance Level

The drought-induced decrease in Δ^13^C_obs_ resulted in ^13^C enriched photoassimilates (higher δ^13^C_nf_ compared to control; **Table 2**), which in turn were transported to the roots and metabolized during SR, as seen in the increase of δ^13^C_SR_. Compared to the reduction of shoot ^13^Δ_obs_ by about 8‰ (i.e., about 31%) during drought, this change in daytime δ^13^C_SR_ of about 1–1.5‰ (i.e., about 5%) was rather small (**Figures 1** and **4**; **Table 1**), probably caused by processes occurring during the transport of recent photoassimilates from leaves to roots. First, the isotopic signal of recently fixed assimilates is often diminished and its temporal variation dampened due to intermediate/transient storage and mixing of different carbon pools alongside their pathway from the leaves to the roots (Brüggemann et al., 2011; Gessler et al., 2014). Second, the total root carbon pool (i.e., standing biomass), into which the recently fixed assimilates are mixed, is much larger compared to the amount of carbon in assimilates supplied to the roots. Third, soil CO_2_ eﬄux is composed of several CO_2_ sources, amongst which root respiration is only one. In particular considering the contribution of heterotrophic respiration, the change in the isotopic signal of leaf carbon due to environmental changes should be much less expressed in the isotopic signal of soil CO_2_ eﬄux (Wingate et al., 2010; Casals et al., 2011). These combined processes probably also lead to difficulties to detect any change in δ^13^C_SR_ in the field at natural isotope abundance level after drought. For example, Unger et al. (2010) found only a small increase in δ^13^C_SR_ after drought using a Keeling plot approach, despite a higher relative contribution of root respiration to total soil CO_2_ eﬄux. Nevertheless, we could clearly show the effect of drought on the isotopic composition of SR – using high temporal resolution laser spectroscopy in our study under controlled environmental conditions.

### Above- to Belowground Coupling

We observed a strong relationship between SR and net assimilation, transpiration and stomatal conductance for drought-treated plants during both drought and recovery periods (**Figure 5**). This pointed to a strong coupling of above- to belowground carbon fluxes. However, all aboveground variables were highly correlated and were all strongly related to VPD. Therefore, it was not possible to disentangle if the reduction in available carbohydrates (drought effect on A_N_) and/or a water-limited deceleration of carbohydrates supply to the roots due to the interaction of xylem and phloem flow rates (drought effect on g_s_ and E, Salmon et al., 2014) limited root respiration during drought. Nevertheless, the significant increase in δ^13^C_SR_ due to drought and the subsequent decrease after re-watering directly related to changes in Δ^13^C_obs_, thus providing indirect methodological evidence that recently fixed photoassimilates rather than storage reserves drive root and rhizosphere respiration. Recent pulse-labeling studies demonstrate that beech maintains transport of recently fixed photoassimilates into the roots under moderate drought stress (Blessing et al., 2015; Hommel et al., 2016), which was suggested as strategy to support root functioning via osmotic adjustment (Hommel et al., 2016). Thus, beech saplings continue allocating carbon to the roots even under carbon limited conditions.

However, the drought effect on δ^13^C_SR_ at natural isotope abundance of about 1–1.5‰ in our small-scale model ecosystems is still relatively small in order to be detected in a natural ecosystem, considering the higher level of complexity. In a forest ecosystem, different plant heights and transport velocities of recently fixed photoassimilates cause various time lags for the carbon isotope signal to be translated from above- to belowground tissues. Furthermore, different plant species may suffer from different stress levels. Wingate et al. (2010) published the only study (to the authors knowledge) in which Δ^13^C on a branch level were measured at high resolution simultaneously with δ^13^C_SR_ in the field, confirming that time lagged correlations between the carbon isotope composition of recently fixed photoassimilates and SR might be weakly or even anticorrelated (Wingate et al., 2010).

### Recovery after Re-watering

A fast recovery was observed in Δ^13^C_obs_ as well as in the carbon isotopic signatures of the neutral fraction (δ^13^C_nf_), which represents mainly sugars. Both δ^13^C_nf_ and δ^13^C_ws_ increased during the drought period for drought-treated plants compared to control plants, but after the recovery period, only the water soluble fraction (δ^13^C_ws_) representing several compounds like amino acids, organic acids, and sugars (Gessler et al., 2007a) still showed a drought signal. Since Δ^13^C_obs_ already fully recovered 7 days after re-watering (DOY 122), its isotopic signal was obviously imprinted on δ^13^C_nf_ but not (sufficiently) on δ^13^C_ws_, which integrates over longer time spans – in this study: at least 10 days (δ^13^C_nf_ and δ^13^C_ws_ both sampled on DOY 125).

Although drought caused a stronger reduction in stomatal conductance (-65%) than in net assimilation (-34%) and transpiration (-42%), all variables fully recovered within 4 days after re-watering. Net assimilation recovered fastest, reaching full recovery already after 3 days, similar to results reported by Zang et al. (2014). Reduced stomatal conductance of drought-treated plants remained slightly lower longer into the recovery period compared to net assimilation, as reported in earlier studies (Tognetti et al., 1995; Miyashita et al., 2005; Martorell et al., 2014). The largest contributions toward full recovery in our study occurred within the day of re-watering and particularly during the following day, similar to earlier reports on other plants (kidney bean, Miyashita et al., 2005; pubescent oak, Gallé et al., 2007), which is in line with fast water uptake after re-watering (Volkmann et al., 2016). Thus, the fast initial recovery of aboveground gas exchange variables indicated that beech saplings were relatively resilient to the (moderate) drought experienced and could restore their carbon and water fluxes within a few days. Recovery of gas exchange after drought depends on the stress level (Miyashita et al., 2005), which makes it difficult to compare different studies. But also other studies (Blessing et al., 2015; Hommel et al., 2016) indicate the high resilience of beech saplings to moderate drought stress.

## Conclusion

Above- and belowground CO_2_ fluxes were strongly coupled, both during drought and the subsequent recovery period. We showed that young beech saplings recovered within a few days from moderate drought conditions, even though they had drastically reduced stomatal conductance, and therefore, aboveground carbon and water fluxes during drought. This resilient behavior is likely to help beech in Central Europe, the area of dominance for beech in the natural vegetation (Ellenberg, 2010), particularly at young age when their root systems are not yet fully developed, coping with more frequent drought events in the future.

## Author Contributions

CB processed the data. MB designed and conducted the experiment. All authors equally contributed to data interpretation, wrote, and critically revised the manuscript.

## Conflict of Interest Statement

The authors declare that the research was conducted in the absence of any commercial or financial relationships that could be construed as a potential conflict of interest.

## References

[B1] Alstad K. P. Lai C.-T. Flanagan L. B. Ehleringer J. R. (2007). Environmental controls on the carbon isotope composition of ecosystem-respired CO2 in contrasting forest ecosystems in Canada and the USA. *Tree Physiol.* 27 1361–1374. 10.1093/treephys/27.10.1361 17669727

[B2] Barthel M. Hammerle A. Sturm P. Baur T. Gentsch L. Knohl A. (2011). The diel imprint of leaf metabolism on the δ13C signal of soil respiration under control and drought conditions. *New Phytol.* 192 925–938. 10.1111/j.1469-8137.2011.03848.x 21851360

[B3] Barthel M. Sturm P. Hammerle A. Buchmann N. Gentsch L. Siegwolf R. (2014). Soil H218O labelling reveals the effect of drought on C18OO fluxes to the atmosphere. *J. Exp. Bot.* 65 5783–5793. 10.1093/jxb/eru312 25100825

[B4] Blessing C. H. Werner R. A. Siegwolf R. Buchmann N. (2015). Allocation dynamics of recently fixed carbon in beech saplings in response to increased temperatures and drought. *Tree Physiol.* 35 585–598. 10.1093/treephys/tpv024 25877767

[B5] Breda N. Huc R. Granier A. Dreyer E. (2006). Temperate forest trees and stands under severe drought: a review of ecophysiological responses, adaptation processes and long-term consequences. *Ann. For. Sci.* 63 625–644. 10.1051/forest:2006042

[B6] Brüggemann N. Gessler A. Kayler Z. Keel S. G. Badeck F. Barthel M. (2011). Carbon allocation and carbon isotope fluxes in the plant-soil-atmosphere continuum: a review. *Biogeosciences* 8 3457–3489. 10.5194/bg-8-3457-2011

[B7] Burri S. Sturm P. Prechsl U. E. Knohl A. Buchmann N. (2014). The impact of extreme summer drought on the short-term carbon coupling of photosynthesis to soil CO2 eﬄux in a temperate grassland. *Biogeosciences* 11 961–975. 10.5194/bg-11-961-2014

[B8] Casals P. Lopez-Sangil L. Carrara A. Gimeno C. Nogues S. (2011). Autotrophic and heterotrophic contributions to short-term soil CO2 eﬄux following simulated summer precipitation pulses in a Mediterranean dehesa. *Global Biogeochem. Cycles* 25 1–12. 10.1029/2010gb003973

[B9] Cernusak L. A. Ubierna N. Winter K. Holtum J. A. M. Marshall J. D. Farquhar G. D. (2013). Environmental and physiological determinants of carbon isotope discrimination in terrestrial plants. *New Phytol.* 200 950–965. 10.1111/nph.12423 23902460

[B10] Ekblad A. Högberg P. (2001). Natural abundance of 13C in CO2 respired from forest soils reveals speed of link between tree photosynthesis and root respiration. *Oecologia* 127 305–308. 10.1007/s004420100667 28547099

[B11] Ellenberg H. (2010). *Vegetation Mitteleuropas mit den Alpen in ökologischer, dynamischer und historischer Sicht*. Stuttgart: Eugen Ulmer KG.

[B12] Epron D. Bahn M. Derrien D. Lattanzi F. A. Pumpanen J. Gessler A. (2012). Pulse-labelling trees to study carbon allocation dynamics: a review of methods, current knowledge and future prospects. *Tree Physiol.* 32 776–798. 10.1093/treephys/tps057 22700544

[B13] Evans J. R. Sharkey T. D. Berry J. A. Farquhar G. D. (1986). Carbon isotope discrimination measured concurrently with gas exchange to investigate CO2 diffusion in leaves of higher plants. *Aust. J. Plant Physiol.* 13 281–292. 10.1104/pp.111.176495

[B14] Farquhar G. D. O’Leary M. H. Berry J. A. (1982). On the relationship between carbon isotope discrimination and the intercellular carbon dioxide concentration in leaves. *Aust. J. Plant Physiol.* 9 121–137. 10.1111/j.1469-8137.2008.02518.x

[B15] Gallé A. Haldimann P. Feller U. (2007). Photosynthetic performance and water relations in young pubescent oak (*Quercus pubescens*) trees during drought stress and recovery. *New Phytol.* 174 799–810. 10.1111/j.1469-8137.2007.02047.x 17504463

[B16] Gavrichkova O. Proietti S. Moscatello S. Portarena S. Battistelli A. Matteucci G. (2011). Short-term natural δ13C and δ18O variations in pools and fluxes in a beech forest: the transfer of isotopic signal from recent photosynthates to soil respired CO2. *Biogeosciences* 8 2833–2846. 10.5194/bg-8-2833-2011

[B17] Gentsch L. Sturm P. Hammerle A. Siegwolf R. Wingate L. Ogee J. (2014). Carbon isotope discrimination during branch photosynthesis of *Fagus sylvatica*: field measurements using laser spectrometry. *J. Exp. Bot.* 65 1481–1496. 10.1093/jxb/eru024 24676031

[B18] Gessler A. Ferrio J. P. Hommel R. Treydte K. Werner R. A. Monson R. K. (2014). Stable isotopes in tree rings: towards a mechanistic understanding of isotope fractionation and mixing processes from the leaves to the wood. *Tree Physiol.* 34 796–818. 10.1093/treephys/tpu040 24907466

[B19] Gessler A. Keitel C. Kodama N. Weston C. Winters A. J. Keith H. (2007a). δ13C of organic matter transported from the leaves to the roots in *Eucalyptus delegatensis*: short-term variations and relation to respired CO2. *Funct. Plant Biol.* 34 692–706. 10.1071/fp07064 32689397

[B20] Gessler A. Keitel C. Kreuzwieser J. Matyssek R. Seiler W. Rennenberg H. (2007b). Potential risks for European beech (*Fagus sylvatica* L.) in a changing climate. *Trees* 21 1–11. 10.1007/s00468-006-0107-x

[B21] Göttlicher S. Knohl A. Wanek W. Buchmann N. Richter A. (2006). Short-term changes in carbon isotope composition of soluble carbohydrates and starch: from canopy leaves to the root system. *Rapid Commun. Mass Spectrom.* 20 653–660. 10.1002/rcm.2352 16444688

[B22] Granier A. Reichstein M. Breda N. Janssens I. A. Falge E. Ciais P. (2007). Evidence for soil water control on carbon and water dynamics in European forests during the extremely dry year: 2003. *Agric. For. Meteorol.* 143 123–145. 10.1016/j.agrformet.2006.12.004

[B23] Grimm V. Wissel C. (1997). Babel, or the ecological stability discussions: an inventory and analysis of terminology and a guide for avoiding confusion. *Oecologia* 109 323–334. 10.1007/s004420050090 28307528

[B24] Högberg P. Nordgren A. Buchmann N. Taylor A. F. S. Ekblad A. Högberg M. N. (2001). Large-scale forest girdling shows that current photosynthesis drives soil respiration. *Nature* 411 789–792. 10.1038/35081058 11459055

[B25] Hommel R. Siegwolf R. Zavadlav S. Arend M. Schaub M. Galiano L. (2016). Impact of interspecific competition and drought on the allocation of new assimilates in trees. *Plant Biol.* 18 785–796. 10.1111/plb.12461 27061772

[B26] Keitel C. Adams M. A. Holst T. Matzarakis A. Mayer H. Rennenberg H. (2003). Carbon and oxygen isotope composition of organic compounds in the phloem sap provides a short-term measure for stomatal conductance of European beech (*Fagus sylvatica* L.). *Plant Cell Environ.* 26 1157–1168. 10.1046/j.1365-3040.2003.01040.x

[B27] Knohl A. Werner R. A. Brand W. A. Buchmann N. (2005). Short-term variations in δ13C of ecosystem respiration reveals link between assimilation and respiration in a deciduous forest. *Oecologia* 142 70–82. 10.1007/s00442-004-1702-4 15378343

[B28] Kuptz D. Fleischmann F. Matyssek R. Grams T. E. E. (2011). Seasonal patterns of carbon allocation to respiratory pools in 60-yr-old deciduous (*Fagus sylvatica*) and evergreen (*Picea abies*) trees assessed via whole-tree stable carbon isotope labeling. *New Phytol.* 191 160–172. 10.1111/j.1469-8137.2011.03676.x 21395596

[B29] Kuzyakov Y. Gavrichkova O. (2010). Time lag between photosynthesis and carbon dioxide eﬄux from soil: a review of mechanisms and controls. *Glob. Change Biol.* 16 3386–3406. 10.1111/j.1365-2486.2010.02179.x

[B30] Martorell S. Diaz-Espejo A. Medrano H. Ball M. C. Choat B. (2014). Rapid hydraulic recovery in *Eucalyptus pauciflora* after drought: linkages between stem hydraulics and leaf gas exchange. *Plant Cell Environ.* 37 617–626. 10.1111/pce.12182 23937187

[B31] McDowell N. G. Bowling D. R. Bond B. J. Irvine J. Law B. E. Anthoni P. (2004). Response of the carbon isotopic content of ecosystem, leaf, and soil respiration to meteorological and physiological driving factors in a *Pinus ponderosa* ecosystem. *Global Biogeochem. Cycles* 18 GB1013–GB1013. 10.1029/2003gb002049

[B32] Merchant A. Peuke A. D. Keitel C. Macfarlane C. Warren C. R. Adams M. A. (2010). Phloem sap and leaf delta C-13, carbohydrates, and amino acid concentrations in *Eucalyptus globulus* change systematically according to flooding and water deficit treatment. *J. Exp. Bot.* 61 1785–1793. 10.1093/jxb/erq045 20211969 PMC2852667

[B33] Miyashita K. Tanakamaru S. Maitani T. Kimura K. (2005). Recovery responses of photosynthesis, transpiration, and stomatal conductance in kidney bean following drought stress. *Environ. Exp. Bot.* 53 205–214. 10.1016/j.envexpbot.2004.03.015

[B34] Nelson D. D. McManus J. B. Herndon S. C. Zahniser M. S. Tuzson B. Emmenegger L. (2008). New method for isotopic ratio measurements of atmospheric carbon dioxide using a 4.3 μm pulsed quantum cascade laser. *Appl. Phys. B* 90 301–309. 10.1007/s00340-007-2894-1

[B35] Pan Y. Birdsey R. A. Fang J. Houghton R. Kauppi P. E. Kurz W. A. (2011). A large and persistent carbon sink in the world’s forests. *Science* 333 988–993. 10.1126/science.1201609 21764754

[B36] Pinheiro J. Bates D. DebRoy S. Sarkar D. Team R. C. (2016). *nlme: Linear and Nonlinear Mixed Effects Models. Version 3*. Available at: http://CRAN.R-project.org/package=nlme

[B37] Plain C. Gerant D. Maillard P. Dannoura M. Dong Y. W. Zeller B. (2009). Tracing of recently assimilated carbon in respiration at high temporal resolution in the field with a tuneable diode laser absorption spectrometer after in situ 13CO2 pulse labelling of 20-year-old beech trees. *Tree Physiol.* 29 1433–1445. 10.1093/treephys/tpp072 19797042

[B38] R Development Core Team (2008–2010). *A Language and Environment for Statistical Computing.* Vienna: R Development Core Team.

[B39] Rascher K. G. Maguas C. Werner C. (2010). On the use of phloem sap δ13C as an indicator of canopy carbon discrimination. *Tree Physiol.* 30 1499–1514. 10.1093/treephys/tpq092 21071770

[B40] Reichstein M. Bahn M. Ciais P. Frank D. Mahecha M. D. Seneviratne S. I. (2013). Climate extremes and the carbon cycle. *Nature* 500 287–295. 10.1038/nature12350 23955228

[B41] Salmon Y. Barnard R. L. Buchmann N. (2014). Physiological controls of the isotopic time lag between leaf assimilation and soil CO2 eﬄux. *Funct. Plant Biol.* 41 850–859. 10.1071/FP13212 32481039

[B42] Scartazza A. Moscatello S. Matteucci G. Battistelli A. Brugnoli E. (2015). Combining stable isotope and carbohydrate analyses in phloem sap and fine roots to study seasonal changes of source-sink relationships in a Mediterranean beech forest. *Tree Physiol.* 35 829–839. 10.1093/treephys/tpv048 26093372

[B43] Schär C. Vidale P. L. Luthi D. Frei C. Haberli C. Liniger M. A. (2004). The role of increasing temperature variability in European summer heatwaves. *Nature* 427 332–336. 10.1038/nature02300 14716318

[B44] Sturm P. Knohl A. (2010). Water vapor δ2H and δ18O measurements using off-axis integrated cavity output spectroscopy. *Atmos. Meas. Tech.* 3 67–77. 10.1002/rcm.7714

[B45] Tognetti R. Johnson J. D. Michelozzi M. (1995). The reponse of European beech (*Fagus sylvatica* L.) seedlings from two Italian populations to drought and recovery. *Trees* 9 348–354. 10.1007/BF00202499

[B46] Unger S. Maguas C. Pereira J. S. Aires L. M. David T. S. Werner C. (2010). Disentangling drought-induced variation in ecosystem and soil respiration using stable carbon isotopes. *Oecologia* 163 1043–1057. 10.1007/s00442-010-1576-6 20217141

[B47] Volkmann T. H. M. Haberer K. Gessler A. Weiler M. (2016). High-resolution isotope measurements resolve rapid ecohydrological dynamics at the soil–plant interface. *New Phytol.* 210 839–849. 10.1111/nph.13868 26864434

[B48] von Caemmerer S. Farquhar G. D. (1981). Some relationships between the biochemistry of photosynthesis and the gas exchange of leaves. *Planta* 153 376–387. 10.1007/BF00384257 24276943

[B49] Werner C. Unger S. Pereira J. S. Maia R. David T. S. Kurz-Besson C. (2006). Importance of short-term dynamics in carbon isotope ratios of ecosystem respiration (delta C-13(R)) in a Mediterranean oak woodland and linkage to environmental factors. *New Phytol.* 172 330–346. 10.1111/j.1469-8137.2006.01836.x 16995920

[B50] Werner R. A. Brand W. A. (2001). Referencing strategies and techniques in stable isotope ratio analysis. *Rapid Commun. Mass Spectrom.* 15 501–519. 10.1002/rcm.258 11268135

[B51] Wingate L. Ogee J. Burlett R. Bosc A. Devaux M. Grace J. (2010). Photosynthetic carbon isotope discrimination and its relationship to the carbon isotope signals of stem, soil and ecosystem respiration. *New Phytol.* 188 576–589. 10.1111/j.1469-8137.2010.03384.x 20663061

[B52] Zang U. Goisser M. Grams T. E. E. Haberle K. H. Matyssek R. Matzner E. (2014). Fate of recently fixed carbon in European beech (*Fagus sylvatica*) saplings during drought and subsequent recovery. *Tree Physiol.* 34 29–38. 10.1093/treephys/tpt110 24420388

